# Hematological Malignancies With Multiple Primary Cancers: A Rare Case Presentation

**DOI:** 10.1155/crh/1527548

**Published:** 2026-01-30

**Authors:** Soudamini Mahapatra, Priyanka Samal, Ashutosh Samal, Tushar Pandey, Naqash Suse, Bhavani Mandava

**Affiliations:** ^1^ Department of Clinical Hematology, Institute of Medical Sciences & Sum Hospital, Siksha ‘O’ Anusandhan Deemed to be University, Bhubaneswar, Odisha, 751003, India, soa.ac.in; ^2^ Department of Cardiology, Institute of Medical Sciences & Sum Hospital, Siksha ‘O’ Anusandhan Deemed to be University, Bhubaneswar, Odisha, 751003, India, soa.ac.in

**Keywords:** metachronous primary malignancy, multiple primary malignancy, synchronous primary malignancy

## Abstract

**Background:**

Two or more primary cancers that arise in two different patients are referred to as multiple primary cancers. We record those cases because optimal therapy requires interdisciplinary cooperation.

**Case 1:**

A 53‐year‐old male presented with intermittent hematuria for one year, fever, burning micturition, appetite loss, and a 3 kg weight loss over 2 months. His CBC showed 81% atypical cells, and bone marrow aspiration and flow cytometry indicated Precursor B‐ALL. He started on the BFM‐2002‐protocol but had persistent hematuria. The USG of the whole abdomen revealed a urinary bladder mass. TURBT and histopathology confirmed low‐grade, noninvasive papillary urothelial neoplasm. Thus, he was diagnosed with Precursor B‐ALL and Low‐Grade Papillary Urothelial Neoplasm Noninvasive.

**Case 2:**

A 53‐year‐old male with a history of anaplastic oligodendroglioma (diagnosed in 2022) presented to the emergency with altered sensorium, headache, and convulsions. He had received radiotherapy and chemotherapy for the past year. In December 2023, he experienced convulsions again due to a recurrence of the oligodendroglioma. His CBC showed an increasing total leukocyte count, reaching 100,000 over five months. Bone marrow and molecular studies indicated a myeloproliferative neoplasm, specifically chronic myeloid neoplasm (CMN) in the chronic phase, with BCR‐ABL1 p210 positive. He was diagnosed with recurrent anaplastic oligodendroglioma (WHO Grade 3) and CMN in the chronic phase.

**Conclusion:**

An increased prevalence of second primary malignancy is anticipated due to the rising cancer burden and the careful screening of index initial malignancy throughout therapy. Determining the best course of action requires careful staging of the cancer and discussion by a multidisciplinary team.

## 1. Introduction

When a patient has two or more primary malignancies, it is referred to as multiple primary malignancies (MPMs) [1]. The most prevalent kind is double primary cancer, which can be either synchronous (SC) or metachronous (MC). SC is the second primary tumor that is discovered concurrently with or within six months of the first primary tumor’s diagnosis [2]. The fast advancement of medical technology and longer life expectancy has been linked to an increased incidence of MPM [3, 4]. The environment, iatrogenic factors, and genetic changes may all be contributing factors to MPMs [5].

In patients with MP tumors and hematologic malignancies, therapy‐related myeloid neoplasm (T‐MN) may be mistaken for a second main cancer. A late side effect of cytotoxic therapies like radiation and chemotherapy is T‐MN. The fourth edition of the WHO diagnostic criteria states that it includes AML, myelodysplastic neoplasms (MDS), and myelodysplastic/myeloproliferative neoplasms (MDS/MPN) but not lymphoblastic leukemia or myeloproliferative neoplasm (MPN). Instead of calling it “T‐MN,” the most recent fifth version of the WHO diagnostic criteria suggests calling it “myeloid neoplasm postcytotoxic therapy” [6]. Cytogenetic and molecular profiling demonstrates distinct biological differences between T‐MN and de novo myeloid neoplasms, reflecting divergent pathogenetic mechanisms. Complex karyotypes and other high‐risk cytogenetic abnormalities are more prevalent in instances connected to therapy. Additionally, next‐generation sequencing (NGS) has revealed a mutational profile that is largely comparable to de novo cases but shows a higher prevalence of mutations in poor prognostic genes such as TP53, SETBP1, and SRSF2, along with a lower incidence of mutations in genes including NPM1, FLT3, and IDH1/2 [7].

Because of the rising cancer burden and close monitoring of index primary malignancy (IPM) during treatment, a rise in the incidence of second primary malignancy (SPM) is expected. Because synchronous MPMs (SMPMs) and metachronous MPMs (MMPMs) are uncommon, we offer two examples.

## 2. Clinical History

### 2.1. Case 1

A 53‐year‐old male presented with hematuria with passage of clots intermittently for 1 year, loss of appetite and 3 kg weight loss over 2 months, fever, and burning micturition for 15 days. He visited the hematology OPD, and routine investigations showed Hb‐14.0, TLC‐41.57, TPC‐10k‐20 k/cmm, N‐7, L‐12, atypical cells‐81%. Other routine investigations were normal. He then underwent bone marrow aspiration and biopsy, which was suggestive of Acute Lymphoblastic Leukemia (ALL) (92% blasts, MPO negative, PAS block positivity, and ALL‐L2). Flow cytometry confirmed Pre‐B‐ALL (CD34, nuTDT, CD19, and cyCD79a positive). Conventional cytogenetics showed 50–55XY, +X, +2, +4, +6, t (9; 22) (q34, q11; 2), +11, +14, +21, der (22) t (9; 22) [cp15]. RT‐PCR for BCR‐ABL1 was positive for P190 minor transcript [IS%]‐25.16764949. Based on these reports, he was started on the BFM2002 protocol for ALL.

**Figure 1:**
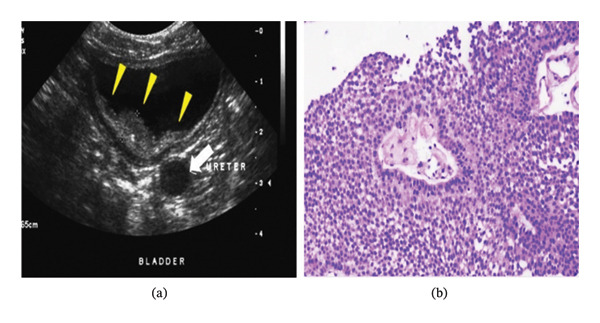
(a) USG of the abdomen and pelvis reveals an intraluminal mass that extends up to the ipsilateral VUJ (TCC) and involves the right posterolateral wall of the bladder. (b) HP (low power 10 ×): low‐grade papillary urothelial carcinoma, noninvasive.

Hematuria was initially thought to be due to a low platelet count, so he was transfused with 6 units of RDP. Despite increased platelet counts, he still had hematuria with passage of clots. An ultrasonography of the whole abdomen and pelvis was done, which revealed an intraluminal mass involving the right posterolateral wall of the urinary bladder extending up to the ipsilateral vesicoureteric junction (VUJ) (possible transitional cell carcinoma (TCC)) (Figure 1(a)). As per the urology consultation, CT‐urogram was done, which confirmed a lobulated, heterogeneously enhancing intraluminal mass. A transurethral resection of bladder tumor (TURBT) sample was taken and sent for histopathology (HP) study. HP showed low‐grade, noninvasive papillary urothelial carcinoma (Figure 1(b)).

Accordingly, the patient was diagnosed with precursor B‐cell acute lymphoblastic leukemia (Precursor B‐ALL) and a low‐grade, noninvasive papillary urothelial neoplasm. Following an interdisciplinary discussion, it was decided that, given the indolent nature of the urothelial lesion, surveillance with cystoscopy every three months would be appropriate. Treatment for Precursor B‐ALL was initiated using the BFM 2002 protocol.

### 2.2. Case 2

A 53‐year‐old male presented to the emergency room with altered sensorium, headache, and four episodes of convulsions over the past two days. He has a known history of anaplastic oligodendroglioma (diagnosed in 2022), Type 2 diabetes mellitus, and hypertension (on medications for 10 years).

In December 2022, he first presented with seizures and altered sensorium. An MRI of the brain showed a space‐occupying lesion in the right temporal region, and he underwent surgery on December 1, 2022. The biopsy confirmed anaplastic oligodendroglioma, WHO Grade 3 (IHC: IDH1 strongly positive, GFAP focally positive, P53 negative, S100 strongly positive, and Ki67%‐30%). He received 30 sessions of radiotherapy from January 19, 2023, to March 2023, and was on Tab temozolomide five tablets per month until January 2024. The hematological parameters have been described in Table 1.

**Table 1:** Different hematological parameters of the patient.

Date	20/4/22	2/12/22	24/3/23	28/11/23	5/4/24	8/4/24
Hb	11.1	11.7	13.5	13.4	12.9	12.0
TLC	95.50	4.53	46.04	19.58	53.38	86.05
TPC	158	198	291	245	251	259
N	N + B − 53	68%	81.3	63.1	62.5	92.4
L	7	28%	4.8	11.6	5.2	6.0
M	0	2%	6.2	6.4	5.5	1.2
B	0	—	0.7	1.9	1.0	0.3
E	2	2%	7.0	17.0	5.8	0.1
IG	26.1	—	16.8	—	20	22.2
sBlasts	1	—	—	—	—	—

He was apparently stable for a year, but in December 2023, he began experiencing convulsions again. Consequently, he underwent repeat craniotomy, tumor resection, duraplasty, and cranioplasty on April 24 and was started on steroids. Routine investigations revealed increasing total leukocyte counts, leading to a hematology consultation. Initially suspected to be steroid‐induced or a leukemoid reaction, the TLC continued to rise. Bone marrow aspiration and biopsy indicated a MPN, likely chronic myeloid leukemia in the chronic phase. A molecular study (MPN reflex panel) was positive for BCR‐ABL1 p210. So, he was diagnosed with recurrent anaplastic oligodendroglioma, WHO Grade 3, and chronic myeloid leukemia in the chronic phase. He was started on Tab imatinib 400 mg and is currently stable.

## 3. Discussion

MPM is the term used when a patient develops two or more primary malignant tumors. Malignant tumors are most common around the age of 50, which is also when they occur. It is widely acknowledged that a variety of factors, such as better clinical treatment techniques, genetic defects, environmental problems, lowered immune levels caused by tumors or treatments, longer life expectancy, and genetic defects, are responsible for the increasing incidence of multiple cancers. The prevalence of MPMs varied from 0.73% to 11.7%, according to a review of the literature involving 1,104,269 cancer patients. Both SC and MC MPMs are possible; SMPMs are more prevalent than MMPMs [8].

In such cases, the two malignancies may be diagnosed either simultaneously (SC) or at different time points (MC). The Surveillance, Epidemiology, and End Results (SEER) program defines SMPMs as two or more, first primary cancers diagnosed within two months [9]. In contrast, the International Association of Cancer Registries and the International Agency for Research on Cancer (IACR/IARC) classify SC tumors as diagnosed within a 6‐month period. Malignancies diagnosed beyond these intervals are considered MC [10].

The more widely accepted diagnostic criteria for different types of cancer are the Warren and Gates criteria [1]:

(i) All tumors must be histologically malignant; (ii) each tumor must have unique clinical features; (iii) cancers must occur in multiple organs or locations; (iv) people who have mutual metastases or recurrence are excluded; and (v) the diagnosis of MPMs in the same patient is becoming more frequent than anticipated due to the aging population and the quick advancement of anticancer treatments. Although research in this field is growing, the tumorigenetic pathways of MPMs are yet unknown. MPMs are caused by several factors, including aging, poor lifestyles, cancer treatments, and their interactions.

Two cases of multiple primary cancers are presented here. SC MPN, which happens when two carcinomas are found in the same person at the same time, is exemplified by the first case. MC MPN, which happens when the second malignancy appears six months after the first, is exemplified by the second case. The purpose of presenting these case reports is to increase awareness of the frequency of MPNs and the challenges associated with their treatment.

While environmental and genetic variables influence both B‐ALL and urothelial neoplasms, the genetic changes and pathways involved differ. Both B‐ALL and urothelial cancer express CD10. CD10 expression in urothelial carcinoma is associated with the tumor’s grade, stage, and possibly invasion and metastasis, even though it is a well‐known marker of B‐ALL. The genetic and cytogenetic changes associated with low grade papillary urothelial carcinoma include chromosome 9 deletion, TERT promoter mutations, and FGFR3 mutations [11]. Tumors that express TP53 nuclearly are linked to early onset illness (age < 45).

Somatic mutations in isocitrate dehydrogenase genes (IDH1 and IDH2), initially identified in central nervous system malignancies, have also been reported in primary acute myeloid leukemia (AML) and secondary AML (sAML) arising from MPN but are typically absent in MPN during the chronic phase (CP). Furthermore, mutations involving JAK2, CBL, CBLB, TET2, ASXL1, IDH1, and IDH2 have been identified across a spectrum of myeloid malignancies, including MDS/MPN, MPN, and sAML. Based on the hypothesis that these genes have undergone a comprehensive mutational study in CML. Advance phase (AP) and blast phase (BP) from CML may arise because of these modifications. Unlike the BCR‐ABL1 translocation, which is the hallmark of the disease, IDH mutations are not thought to be the main cause of CML. They may, however, also aid in the illness’s progression and entry into the blast phase. The mechanisms by which temozolomide cause secondary leukemia are distinct from the genetic changes that underlie CML. The primary cause of CML is the BCR‐ABL1 fusion gene, while temozolomide‐induced secondary leukemia is caused by DNA damage and subsequent mutations [12]. Because these mutations confer an enzymatic gain of function leading to increased production of 2‐hydroxyglutarate, heterozygous acquisition of IDH family mutations may be sufficient to drive malignant transformation. This observation suggests that IDH mutations play a contributory role in the evolution toward a more aggressive phenotype [13]. According to the WHO Classification of Haematolymphoid Tumours, 5^th^ edition, alterations in TP53, RB1, MYC, CDKN2A (p16^INK4a^), NRAS, KRAS, RUNX1 (AML1), TET2, CBL, ASXL1, IDH1, and IDH2 have been documented in transformed stages; however, the precise mechanisms by which these genetic abnormalities drive disease progression remain incompletely understood [14].

The pathogenic type, each tumor’s stage, and the patient’s physical state all influence the therapy strategy for MPMs. Each tumor should be assessed and staged separately when MPMs have been pathologically verified. In certain situations, a baseline PET‐CT may assist identifying several cancers and develop a treatment strategy. Multidisciplinary Team (MDT) discussions are essential for problems that are unsolvable. In general, the tumor that poses the greatest risk to the patient’s survival or quality of life ought to be given priority. Resection of both tumors should be the top goal if surgery is appropriate for the patient with MPMs. It may be used in conjunction with endocrine therapy, chemoradiotherapy, or other treatments as needed.

The National Comprehensive Cancer Network’s (NCCN) recommendations for each tumor’s pathological stage are often followed for treating MPMs. A multidisciplinary team should be involved in the effective evaluation of patients with MPMs. They ought to be properly restaged and handled appropriately.

## 4. Conclusion

Patients suspected of having an MPM should have a comprehensive physical examination to promptly detect and treat any additional potential cancers. Treatment for many cancers starts with developing a personalized treatment strategy for each patient depending on their situation. This covers all‐encompassing therapies such radiation, chemotherapy, surgery, and targeted therapy. There is presently no standardized treatment plan for multiple cancers. These clinical scenarios are rare and pose significant therapeutic challenges, particularly when patients are considered for hematopoietic stem cell transplantation, where management becomes increasingly complex and necessitates close interdisciplinary collaboration. Further research is warranted, especially to optimize treatment strategies for patients with SC or MC MPMs. Improved characterization of the impact of prior therapies on prognosis, antitumor efficacy, and treatment‐related toxicity is also needed.

**Nomenclature**
CMN: Chronic myeloid neoplasm
MPMs: Multiple primary malignancies
T‐MN: Therapy‐related myeloid neoplasm
MDS/MPN: Myelodysplastic/myeloproliferative neoplasms
NGS: Next‐generation sequencing
SPM: Second primary malignancy
IPM: Index primary malignancy
ALL: Acute lymphoblastic leukemia
SMPMs: Synchronous MPMs
MMPMs: Metachronous MPMs
VUJ: Vesicoureteric junction
TURBT: Transuretheral resection of bladder tumor
TCC: Transitional cell carcinoma
NCCN: National Comprehensive Cancer Network
MDT: Multidisciplinary team
sAML: Secondary acute myeloid leukemia
IACR: International Association of Cancer Registries
IARC: International Agency for Research on Cancer
SEER: Surveillance Epidemiology and End Results

## Author Contributions

Soudamini Mahapatra and Priyanka Samal were major contributors to the writing of the manuscript. Ashutosh Samal was responsible for the revision of the article. Tushar Pandey conducted a detailed analysis of the results of imaging examinations. Naqash Suse and Bhavani Mandava supervised the writing of the article and provided a critical review.

## Funding

This research received no specific grant from any funding agency in the public, commercial, or not‐for‐profit sectors.

## Disclosure

The authors have no affiliation with or involvement in any organization or entity with any financial interest in the subject matter or materials discussed in this manuscript.

All authors read and approved the final manuscript.

## Ethics Statement

This case report does not need any ethical approval.

## Consent

The participants provided written informed consent prior to participating in the study.

## Conflicts of Interest

The authors declare no conflicts of interest.

## Data Availability

The data supporting the findings of this study are available from the corresponding author upon reasonable request.
